# Supplementary material for: Exploring health-seeking behavior for non-communicable chronic conditions in northern Bangladesh
Source: PLOS Glob Public Health. 2022 Jun 10;2(6):e0000497. doi: 10.1371/journal.pgph.0000497 (PMC10022368; doi:10.1371/journal.pgph.0000497)
Supplement: S1 Guideline — (DOCX) [file pgph.0000497.s001.docx]

**Semi-structured guideline for in-depth interview with respondent affected by NCD**

**Name of the study: In-depth exploration of health-seeking behavior of Non-Communicable Diseases (NCDs) in Northern Bangladesh**

| **Name of the Interviewer:**  **Date:**  **Day:**  **Union:**  **Sub-district: Mithapukur** | **Time of start:**  **Interview number:**  **Respondent details:**  **Age:**  **Sex:**  **Education:**  **Occupation:**  **Marital status:** |
| --- | --- |

**1. Introductory exchange**

Salam/ Adab. Thank you so much for agreeing to participate in this interview. I am very interested in learning about your experience and thoughts about long term illness which are not contagious. I would like to mention again that your participation is voluntary. Therefore you can choose to stop the interview at any time if you want. All your responses will be recorded, but will be kept confidential. I will keep the interview anonymous, which means that the information I collect from you will not be used anywhere with your name and identifiable information.

Before we start, do you have any questions for me?

**Observe during interview:**

Body language, Facial expression, non-verbal clues, and subtle meanings/gestures, be acutely aware of the context and activity going around.

**2. Opening up questions:**

How are you? / How are you feeling today?

**3. Individual perspectives and experience on own condition**

How would you rate your current health status? (Probe: good, medium, and bad)

Why do you think so?

Are you satisfied/ happy with your current health status? Why?

What are the conditions/ health problems that you have at present?

(listen for specific illness names or local terms like hypertension/*high pressure*, asthma/COPD/*hapani*, diabetes, chronic joint pain/arthritis/*bat jor/giray giray betha bedna*, chronic GI disorder/*gastric*/*Gas er somossha*, physical disability/*pongu/hat pa ochol*)

**4. Detailed Questions:**

How did you first come to know about that/those illnesses? From whom did you learn about it?

Where did you first seek care for this illness? Why?

How do you perceive this illness in terms of severity? (Severe/Medium Severe/Not Severe)

Why?

Can you tell me the places where care for your condition is available? Where do you usually go for this condition? Why?

What have you done so far for this illness? What are you doing now for this illness? (probe: shift between different kinds of providers)

Could you describe your experience about taking care last time? (Probe: When and where did you seek care/take any step for this condition last time?)

How did your experience regarding this illness change your views/ways of taking care of your condition?

Which type of care/provider do you currently prefer the most? Why? (Probes: Cost, quality of care, satisfaction with services, trust, relation with provider)

If type of last sought care and current ongoing treatment are different: Why are you taking a different treatment now? How did you come to this decision?

Can you share with me an important experience that you had while seeking care for this disease that affected the way you take care of this condition now? (Probe: positive/negative experience)

In terms of making a decision to seek care for your illness, how important is cost?

Would your preference be different in terms of quality of care (by quality of care I mean did you like the treatment provided, and did you think that treatment worked?)? Why so?

How did your own characteristics affect the way you approached this condition?

Probe: Gender, age, marital status, religion, education, occupation, co-morbidities

Example: How does your gender affect the way that you take care of your condition?

From your experience of living with this condition, can you mention the most important factors in favor of you to seek care?

Can you tell me about the most important factors that created barriers for you when seeking care for this long-term illness?

**5. Role of Household Characteristics and Contextual factors**

What role did your household and household members play in seeking health care for this condition?

(probes: permission from husband, household approval, autonomy in taking own decision, accompanying person, economic status, other people ill in family)

Do you have any other family members suffering from long-term illness like you? How does it influence your own behavior on your condition?

In what ways did your community and neighbors have a role in the steps you took for this disease?

How does your locality affect the way you seek care for your condition? Can you explain further? (transport, availability of health services/providers, seasonal food insecurity/monga, rainy season affecting roads)

In your opinion, what could be done to make health services for these conditions easily available to people like you?

Do you want to share anything about this illness from your experience?

Thank you for sharing your experience with us. I hope with your experience and thoughts, health care services for people with CNCDs will be improved.

Do you have any questions you would like to ask?

Time of Ending Interview:
