# Supplementary material for: Exploring health-seeking behavior for non-communicable chronic conditions in northern Bangladesh
Source: PLOS Glob Public Health. 2022 Jun 10;2(6):e0000497. doi: 10.1371/journal.pgph.0000497 (PMC10022368; doi:10.1371/journal.pgph.0000497)
Supplement: S2 Guideline — (DOCX) [file pgph.0000497.s002.docx]

**Semi-structured guideline for in-depth interview with healthcare provider**

**Name of the study: In-depth exploration of health-seeking behavior of Non-Communicable Diseases (NCDs) in Northern Bangladesh**

| **Name of the Interviewer:**  **Date:**  **Day:**  **Union:**  **Sub-district: Mithapukur** | **Time of start:**  **Interview number:**  **Respondent details:**  **Age:**  **Sex:**  **Education:**  **Designation:**  **Institute:** |
| --- | --- |

**1. Introductory exchange**

Salam/ Adab. Thank you so much for agreeing to participate in this interview. I am very interested in learning about your experience and thoughts about long term illness/ non-communicable illness (if doctor). I would like to mention again that your participation is voluntary. Therefore, you can choose to stop the interview at any time if you want. All your responses will be recorded, but will be kept confidential. I will keep the interview anonymous, which means that the information I collect from you will not be used anywhere with your name and identifiable information.

Before we start our interview, do you have any questions for me?

**Observe during interview:**

Body language, facial expression, non-verbal clues, and subtle meanings/gestures, be acutely aware of the context and activity going around.

**2. Interview guidelines:**

1. How are you today? Is it a busy day for you?
2. How many patients usually come to you every day/ what types of patients come to you every day?
3. Among the patients who come to you, what is the tentative proportion of people who have long term illnesses?
4. Which long-term illness do they commonly come with?

(Listen for specific illness like hypertension, asthma/COPD, diabetes, chronic joint pain/arthritis, chronic GI disorder/Gastric, physical disability, cancer)

1. At which stage of these diseases do they usually come (early/ advanced stage)?
2. Why do you think they have this practice?
3. (If provider talks about delay in care seeking): where/ in which stage are most of the delay and why?
4. Which kinds of long-term illness do you/ your institute provide services for?
5. What kind of services do you/your facility provide for those diseases?
6. To what extent are people aware that you have these services available for chronic illness?
7. To what extent do people follow your advice and treatment?
8. To what extent do people of this community understand the severity/consequences of having NCDs?
9. In your view, how much need are you being able to meet with your services compared to the actual need of healthcare for NCDs? Why do you think so?
10. From your experience, where do you think majority of people suffering from this condition in this area first seek care from? Why does that happen?
11. From your experience, why do you think people suffering from NCDs decide to seek care from you?
12. From your view, how do you think social norms affect the people suffering from long-term illness take their steps for seeking health care? (Permission from husband, accompanying person, trust)
13. How does cost of your services affect the people coming to seek care from you for these illnesses?
14. How does your gender affect people with non-communicable diseases coming to you for care?
15. How do their familiarity with you/your facility settings affect their health seeking regarding long-term illnesses?
16. From which socio-economic status do most people come to you for NCD care/long term-illness care?
17. Can you share any experience with people coming to seek NCD care from you (good or bad)?
18. In your opinion, do you think all people in this area have equal access to CNCD care? Why?
19. What could be done to increase access to CNCD healthcare in your opinion?

After interview:

- Do you have any final questions?
- Thank you so much for your time.

Interviewer Reflections on the observations after interview:

Interview ending time:
