# Supplementary material for: Exploring health-seeking behavior for non-communicable chronic conditions in northern Bangladesh
Source: PLOS Glob Public Health. 2022 Jun 10;2(6):e0000497. doi: 10.1371/journal.pgph.0000497 (PMC10022368; doi:10.1371/journal.pgph.0000497)
Supplement: S3 Guideline — (DOCX) [file pgph.0000497.s003.docx]

**Guideline for Focus Group Discussion**

**Name of the study: In-depth exploration of health-seeking behavior of Non-Communicable Diseases (NCDs) in Northern Bangladesh**

| **Name of the Moderator: Fatema Rasul**  **Name of Note taker:**  **Date:**  **Day:**  **Union:**  **Sub-district: Mithapukur** | **Time of start:**  **Discussion number:**  **Number of Participants:**  **Age group: Middle age/Elderly**  **Sex:** |
| --- | --- |

**1. Introductory exchange**

Salam/Adab. Thank you so much for agreeing to participate in this discussion. We are hoping you will have important information to share with us regarding the context, culture and norms of the community in shaping health seeking behavior for long-term illnesses. I want to remind you that participation in this discussion is completely dependant on your choice. You can choose not to answer any question or not to participate at any time. With your permission this discussion will be recorded,and notes will be taken by our note-taker. Your shared information will be valuable for this study to increase access of healthcare for people with long-term illness in Bangladesh.Your names will be kept confidential, and under no circumstances will it be exposed.

This discussion will go on for about an hour. Before starting the discussion, we want to set some rules which you have to follow while participating in the discussion.

1. When someone is saying something, please wait. If you have anything to say, say it after that person has finished.
2. If you have any comments, please raise your hand.
3. Please do not abuse or make fun of anyone in the discussion.
4. Please do not make noise or talk at the same time a person is talking. It distracts every one’s attention.

Before starting the discussion, do you have any questions for me?

**2. Opening up questions:**

What type of illnesses do you see mostly around you?

Among those, which are life-long/long term illnesses?

(listen for specific illness names or local terms like hypertension/*hi pressure*, asthma/COPD/*hapani*, diabetes, chronic joint pain/arthritis/*bat jor/giray giray betha bedna*, chronic GI disorder/*gastric*/*Gas er somossha*, physical disability/*pongu/hat pa ochol*)

Have these illness increased or decreased compared to before? Why do you think so?

Do you have any local names for such kind of illness?

**3. Detailed discussion**

What is the usual practice of seeking care for this kind of illness in this community?

Where do the people of this community mostly go to seek care for these kinds of illness? Why? (Probes: Cost, Relation with provider, trust, norms)

Where else is treatment for this kind of condition available? When is it available?

How is the quality of care of those types of care?

Where is the information for services for this kind of illness available?

Considering a person living in this area, can you mention some advantage and disadvantage of seeking care for these conditions?

How would you rate the transport system/facilities from your community to seek care for these kinds of conditions? Why are you saying so?

Can you tell me how to go to the nearest:

- - Community clinic
  - Traditional healer/Homeopath/Village doctor
  - Upazilla Health Complex
  - Private doctor/ hospital
  - Rangpur medical college hospital

How do community norms create advantage/disadvantage for health care seeking of individuals for this kind of condition?

(Probes: Shy to see provider of opposite gender, permission from husband, accompanying patient, awareness of referring to health provider, giving out loans/help)

How do the community people help out when someone needs assistance for this illness (person/money)?

**4. External factors:**

Do you have problems in this area during rainy season? How does it change the way people seek care for these conditions in that time?

Do you have Monga in your area? If yes, how does your Monga affect the way people seek care for these conditions? Can you explain further?

Are you satisfied with the current care available for long-term illness in this community? Why?

As a community, do you think all people have equal access to long-term illness care? Why?

How is it possible to make services for these illnesses more accessible to people of this community?

After interview:

- Do you have any final questions?
- Thank you so much for your time.

**Time of End of discussion:**

**Reflections after the discussion is over:**
