# Supplementary material for: Exploring health-seeking behavior for non-communicable chronic conditions in northern Bangladesh
Source: PLOS Glob Public Health. 2022 Jun 10;2(6):e0000497. doi: 10.1371/journal.pgph.0000497 (PMC10022368; doi:10.1371/journal.pgph.0000497)
Supplement: Attachment — Submitted filename: Response to reviewers- Mar2.docx [file pgph.0000497.s004.docx]

Journal Requirements:

1. Please provide separate figure files in .tif or .eps format only. Please ensure that all files are under our size limit of 20MB.

For more information about how to convert your figure files please see our guidelines: Once you've converted your files to .tif or .eps, please also make sure that your figures meet our format requirements

**Response:**  We have converted our file to .tif format.

2. Your manuscript is missing the following sections: Results. Please ensure these are present, and in the correct order, and that any references to subheadings in your main text are correct. An outline of the required sections can be consulted in our submission guidelines here: <https://journals.plos.org/globalpublichealth/s/submission-guidelines#loc-parts-of-a-submission>

**Response:**  The manuscript had the results under the name “Findings”. We have changed the heading “Findings” to “Results”.

3. In the online submission form, you indicated that "The qualitative data generated and analyzed during this study are not publicly available due to the data containing information that could compromise research participant’s privacy, but are available on reasonable request. Restrictions may apply.". All PLOS journals now require all data underlying the findings described in their manuscript to be freely available to other researchers, either 1. In a public repository, 2. Within the manuscript itself, or 3. Uploaded as supplementary information.
This policy applies to all data except where public deposition would breach compliance with the protocol approved by your research ethics board. If your data cannot be made publicly available for ethical or legal reasons (e.g., public availability would compromise patient privacy), please explain your reasons by return email and your exemption request will be escalated to the editor for approval. Your exemption request will be handled independently and will not hold up the peer review process, but will need to be resolved should your manuscript be accepted for publication. One of the Editorial team will then be in touch if there are any issues.

**Response:**  All qualitative data relevant to this manuscript have been included as quotes in this manuscript. The transcripts are in Bangla, and they are not publicly available due to them containing information that could compromise research participant’s privacy, but their key quotes have been translated to English and made available in this manuscript. The other relevant information such as the tools for IDIs and FGDs have been included as supplementary information.

4. Please amend your detailed Financial Disclosure statement. This is published with the article, therefore should be completed in full sentences and contain the exact wording you wish to be published.

ii). State the initials, alongside each funding source, of each author to receive each grant.
iii). State what role the funders took in the study. If the funders had no role in your study, please state: “The funders had no role in study design, data collection and analysis, decision to publish, or preparation of the manuscript.”

**Response:**

We will update the information of funding source when uploading the revised manuscript. Costs for field data collection were covered using bonus research funding assigned directly to Prof. Manuela De Allegri by the University Hospital Heidelberg to cover research costs for her team. During data analyses, the corresponding author Fatema Binte Rasul was supported by a sandwich PhD scholarship of USAID’s Next Generation of Public Health Experts Program and a DAAD doctoral degree completion grant under the STIBET program. The funders had no role in the design and conduct of the study; collection, analysis, and interpretation of data; writing of the article; or decision to submit the article for publication.

Additional Editor Comments (if provided):


[Note: HTML markup is below. Please do not edit.]

Reviewers' comments:

Reviewer's Responses to Questions

**Comments to the Author**

1. Does this manuscript meet PLOS Global Public Health’s [publication criteria](https://journals.plos.org/globalpublichealth/s/criteria-for-publication)? Is the manuscript technically sound, and do the data support the conclusions? The manuscript must describe methodologically and ethically rigorous research with conclusions that are appropriately drawn based on the data presented.

Reviewer #1: Yes

Reviewer #2: No

2. Has the statistical analysis been performed appropriately and rigorously?

Reviewer #1: N/A

Reviewer #2: N/A

3. Have the authors made all data underlying the findings in their manuscript fully available (please refer to the Data Availability Statement at the start of the manuscript PDF file)?

The [PLOS Data policy](https://journals.plos.org/globalpublichealth/s/data-availability) requires authors to make all data underlying the findings described in their manuscript fully available without restriction, with rare exception. The data should be provided as part of the manuscript or its supporting information, or deposited to a public repository. For example, in addition to summary statistics, the data points behind means, medians and variance measures should be available. If there are restrictions on publicly sharing data—e.g. participant privacy or use of data from a third party—those must be specified.

Reviewer #1: Yes

Reviewer #2: Yes

4. Is the manuscript presented in an intelligible fashion and written in standard English?

PLOS Global Public Health does not copyedit accepted manuscripts, so the language in submitted articles must be clear, correct, and unambiguous. Any typographical or grammatical errors should be corrected at revision, so please note any specific errors here.

Reviewer #1: Yes

Reviewer #2: Yes

5. Review Comments to the Author

Please use the space provided to explain your answers to the questions above. You may also include additional comments for the author, including concerns about dual publication, research ethics, or publication ethics. (Please upload your review as an attachment if it exceeds 20,000 characters)

Reviewer #1: The manuscript describes a synthesis of health seeking approaches in northern Bangladesh population suffering from NCDs. I believe the importance of this paper stems from the applicability of the approach to a large population base, and the challenge we will have in healthcare delivery. This work presents a paradigm that can be broadly and usefully applied.
It is an important topic, needed, and useful summary of the status of service delivery from a certain perspective. The authors, however, need to be bolder and more analytical. This is an opinion piece and a thorough analysis is needed. For example, the financial status of the population surveyed. Including more unemployed and financially poor will result in bias of behavior. A certain view is implied by the authors, but they could be more explicit.

**Response:**  Thank you for your great suggestion. One of the key reasons that we encountered so many unemployed people were in the sample is because all of the women (14) were unemployed. That being said, such rates of stay-at-home women is normal in the locality of Rangpur. Moreover, the sample had three people who had retired because of age, and one who was unemployed due to paralysis. We have explicitly mentioned of this issue in the limitations section, so that readers are aware of it when looking at the results. Given that 45% of Rangpur’s population falls under the national poverty line, our study sample had very subtle differences among their economic conditions, we have mentioned of such economic condition in the study settings, and have now specified the number of respondents who belonged to the poorer group and who belonged to the less poor group in our manuscript results.

The paper would be both more compelling and useful to a broad readership if the authors moved beyond providing a simple summary of the problems and deeply examined why there is difference in approach in some areas if they included a big sample size with a larger geographical base and then used the evidence they have compiled to suggest a path forward.
I think this oversimplification instigates deeper analysis in other areas as well. I would like to see more examination of the validation requirement beyond the lens of patient health seeking behavior vis a vis availability of resources and healthcare services at the disposal of the segment of population provided by the state in this region.

**Response:**  Thank you for your comment. In the manuscript, we have now tried to address the scarcity of the required resources in health care services, especially in the grassroot levels, and have mentioned that it creates a major barrier in terms of NCD related health-seeking. Our sample, however, does not allow us to explore in a deeper manner geographical differences. While it is true that our sample is relatively large for a qualitative sample, it was geographically clustered and all hence all “dependent” on the same set of facilities. In a prior quantitative analysis, we have been able to quantify the role of selected health system characteristics on individual healthcare seeking behavior.

The authors have tried to capture brilliantly the population beliefs in interview transcripts. The paper relates to in-depth belief of the local populace and thus provide an understanding of the basic problem which can be addressed by the state.

Finally, the takeaway message from this article is well summarized, with suggested directions and immediate challenges to overcome, a call to action and further analysis.

Overall, though this is a timely and needed research. It is well researched and nicely written with rich metaphor. With minor modifications addressing financial status (no details just simplified explanation as below poverty line/ lower middle class, middle class etc. would suffice) this will be a worthwhile review paper. With more significant modification where the authors dig deeper into the complexities and controversies and truly grapple with their implications to suggest a way forward, this could be a very influential paper.

**Response:**  Given that 45% of Rangpur’s population falls under the national poverty line, our study sample had very subtle differences among their economic conditions. We have now added the economic conditions of the respondents as two groups: poorer household, and less poor households the manuscript.

Reviewer #2: Fundamental
1. Why the authors included participants with at least one chronic condition? Why not the healthy individual? The participants with chronic conditions have well orientation with the disease process and management. This orientation suppresses their primary belief and practice. Inclusion of this study group probably overrepresent the study findings/overestimated the all components of analysis.

**Response:**  We have included participants with at least one chronic condition, not a healthy individual because our aim was to find out how a person navigates the health system for their health needs when they are affected by chronic non-communicable disease. This has been a purposive sampling decision. The awareness related to chronic non-communicable diseases is low in Bangladesh, therefore choosing a group who are already dealing with chronic conditions increased our chances to elicit more information related to health-seeking for these conditions. We have now mentioned it explicitly in our manuscript, to prevent misinterpretation by readers as an over-representation of the general population.

2. Where the data was collected? At the healthcare facilities or household or any other sites?

**Response:**  Thank you for your great question. The in-depth interviews with the people affected by chronic non-communicable conditions were mostly conducted at their home, a few in another place that the respondents chose. The interviews with the health-care providers were conducted mostly at their workplace, a couple were done in their home as per their choice, and the focused group discussions were conducted inside the community, in an open common place where 6-8 people could sit and discuss comfortably. This information has now been added to our revised manuscript.

3. This reviewer assessed the questionnaire (S2 file, health provider interview guideline) used for healthcare providers. Question number 05 is really irrational or without logic. The question was “At which stage of these diseases do they usually come (early/ advanced stage)?”. Among the 21 healthcare providers, 18 were semi qualified. How this semi-qualified healthcare providers graded a disease into stages as early or advanced? How the authors segregated the healthcare providers into qualified and semi-qualified? This is a vital question in this study as the findings suggested (aligned with global findings) the health-seeking behaviors initiated when symptoms become sever. The findings related to this question will misguide the readers and subsequent policy recommendation.

**Response:**  By asking the question no. 5 as mentioned above, we aimed to assess the opinions of the local health care providers if the needs to provide public health interventions for people with NCDs were more in people in early stages of their conditions or in the late stages of their conditions. The aim was not to get an exact medical assessment of the early or late stages of people affected with CNCDs, rather it was to identify the provider’s opinions about when people seek out health care, so that the researchers could get an idea about how prompt people are at seeking out care after the symptoms appear in them.

We defined semi-qualified professionals as any allopathic or traditional provider with some degree of training and experience in primary care, but no specific expertise in CNCDs (e.g., medical assistants, village doctors, community health workers, drugstore keepers, and traditional healers). We defined qualified professionals as registered and trained physicians (i.e., MBBS doctors). We have updated our manuscript with this definition.

4. The authors presented “The government health workers confirmed that NCD diagnostic tools and medications are only available at the Upazilla Health Complex…” What percentages of government health workers participated in this study? I have not found any government health workers included in this study group. What the authors mean by ‘Both public and private practice’ as affiliation? Need clarification.

**Response:**  Among the 21 health providers that were interviewed for this study, 8 were government employees. There is a table regarding the characteristics of health providers, where we have mentioned the affiliation of the health providers being either public or private, or both. The government health workers are those who have public practice, and both public and private practice. By both public and private affiliation, we mean that those providers are employees of the public health system, but they also practice privately on their own during their spare hours. This is a common practice in Bangladesh, also known as moonlighting.

5. Who are the actors participated in this study? Inclusion of actors increases the strength of such study. Did the authors include teachers or religious leaders or any representative of a government health facility?

**Response:**  The key informants that we interviewed for this study are the local health providers. We have not interviewed any teachers or religious leaders for this study. We have interviewed a number of healthcare providers of the government health facilities as representatives of government health facilities in those locations.

6. Why the authors avoided to represent their findings based on urban and rural? This is a fundamental issue for a country with fragile health system. Moreover, the health-seeking behaviors largely vary in these two areas. This reviewer like to see the urban-rural variations in terms of ‘health-seeking behaviors.

**Response:**  We have looked at our finding through the urban and rural lens, and have reported about our findings that geographical location is indeed a barrier in seeking healthcare for NCDs. We have reported about it in the findings section, under “contextual factors and health-seeking experience”. We agree with the reviewer that urban and rural health-seeking vary largely, and as such, expecting to find variation between the urban and rural locations, we had purposely sampled half of the participants from urban and the other half from the rural location. We have also reported if the participant belongs to urban or rural location, beside each quote.

7. Did the authors assess ‘Knowledge’ about the term ‘Noncommunicable Diseases’? And what are the factors responsible for NCDs? These are the basics that influenced their spiritual action. Based on the questionnaire the authors used, this reviewer assuming ‘knowledge’ in terms of NCD and its risk factors did not asses explicitly. However, the authors tried to find out the sources (family, friends, and semi-qualified professionals) those shaped their knowledge. How the authors interpret their overall findings in this regard? If the participants did not hear the term ‘Noncommunicable Diseases’ in their lifetime, then how their conception will be built up around this entity of disease?

**Response:**  Thank you for your great questions. Our aim was to assess the health seeking behavior for chronic NCDs and the beliefs and environment that lead to such health seeking behaviors. As such, we did not assess knowledge as deeply as could be assessed, as our primary concern was the health seeking behavior. To avoid this issue of people who never heard of the term “non communicable diseases”, we had used “tracer conditions” in the survey that preceded this study, and categorized them into certain conditions. That is, the data collectors read out to the participants the name of the disease, as well as its symptoms. Chronically ill people often get used to the symptoms due to its persistent presence; and often consider them not so important to report. Administering questionnaire with both diagnosis and symptoms help remind the respondents of the persistent conditions, and increased their ability to verbalize the problems (Kroeger 1983b). Such kind of questionnaire has been used previously in household surveys of low-resource settings, to elicit information about CNCDs (Wang et al. 2015). And from that survey, we only selected individuals who reported of having at least one chronic condition (more than 3 months continuously). In this way, we ensured that we are dealing with people who had reported of having a chronic NCD, to be able to ask about their health-seeking behaviors.

As for the focused group discussions, where community people were asked about their perceptions about NCDs, we led them towards discussing those conditions by asking them to say some names of long-term illnesses, or illnesses that stayed life-long in people, and did not go away. And then the facilitator listened for specific illness names or local terms of long-term conditions that came out through pre-tests, and went ahead and repeated those condition names, and led them into a discussion regarding those conditions (see S3 file-focused group discussion guide).

8. What about the limitations of this study? The authors avoided to mention sampling strategy as purposive, small numbers of workshop and separate workshops for both men and women as anticipated that gender-related differences in care seeking were likely. Moreover, there is a gray area about the engagement of local leaders or actors.

**Response:**  Thank you for your comments. For qualitative studies, purposive sampling is a strength, hence we did not mention it in our limitations. We have specified in the methods section that we have purposely sampled the respondents based on certain aspects. We did not interview or engage any local leader or actor in our study. Before interviewing the government health providers, we had obtained permission from the subdistrict UHNFPO (Upazilla Health and Family Planning Officer). We only interviewed healthcare providers as they would be able to give better information to help us triangulate the health seeking behaviors of the respondents who suffer from NCDs.

Minors
1. Change the title as it is reflecting a specific religious belief. Make it as simple as possible.

**Response:**  Thank you so much for your comment. We have changed and made the title simpler and general: “Exploring health-seeking behavior for non-communicable chronic conditions in northern Bangladesh”

2. Change the reference number 1 & replace with recent GBD study

**Response:**  Thank you so much for your suggestion. We have replaced it with recent GBD study.

3. Authors have not yet defined the term ‘health-seeking behavior’ in their manuscript? What are the components they considered to assess?

**Response:**  Health seeking behavior refers to the series of actions that people undergo with the aim of curing ill-health as per their perception (Ward et al. 1997). For this study, we have focused on the process or pathway of responding to a perceived illness (MacKian 2003).

We have included the definition of health-seeking behavior for this study within the manuscript now.
